# Tuning the allosteric regulation of artificial muscarinic and dopaminergic ligand-gated potassium channels by protein engineering of G protein-coupled receptors

**DOI:** 10.1038/srep41154

**Published:** 2017-02-01

**Authors:** Christophe J. Moreau, Jean Revilloud, Lydia N. Caro, Julien P. Dupuis, Amandine Trouchet, Argel Estrada-Mondragón, Katarzyna Nieścierowicz, Nicolas Sapay, Serge Crouzy, Michel Vivaudou

**Affiliations:** 1 Institut de Biologie Structurale (IBS), 38044 Grenoble, France; 2 IBS, Univ. Grenoble Alpes, 38044 Grenoble, France; 3 IBS, CNRS, LabEx ICST, 38044 Grenoble, France; 4 Laboratoire de Chimie et Biologie des Métaux (LCBM), CEA, Institut de Biosciences et Biotechnologies de Grenoble (BIG), UMR 5249, 38054 Grenoble, France; 5 LCBM, Univ. Grenoble Alpes, 38054 Grenoble, France; 6 LCBM, CNRS, 38054 Grenoble, France

## Abstract

Ligand-gated ion channels enable intercellular transmission of action potential through synapses by transducing biochemical messengers into electrical signal. We designed artificial ligand-gated ion channels by coupling G protein-coupled receptors to the Kir6.2 potassium channel. These artificial channels called ion channel-coupled receptors offer complementary properties to natural channels by extending the repertoire of ligands to those recognized by the fused receptors, by generating more sustained signals and by conferring potassium selectivity. The first artificial channels based on the muscarinic M2 and the dopaminergic D2_L_ receptors were opened and closed by acetylcholine and dopamine, respectively. We find here that this opposite regulation of the gating is linked to the length of the receptor C-termini, and that C-terminus engineering can precisely control the extent and direction of ligand gating. These findings establish the design rules to produce customized ligand-gated channels for synthetic biology applications.

Ligand-gated ion channels or ionotropic receptors constitute a specific family of ion channels characterized by large extracellular ligand-binding domains physically and functionally connected to the transmembrane pore. They play essential roles in numerous physiological functions by translating extracellular biochemical messages into an electrical signal by modulation of the membrane potential. The family of vertebrate LGICs is divided in subgroups according to their sequence similarity and subsequently to their specificity to endogenous ligands. The ligands γ-aminobutyric acid (GABA), glycine (Gly), serotonin, acetylcholine and zinc are recognized by members of the Cys-loop subgroup, while glutamate and ATP are recognized by the glutamate and the P2X receptors, respectively. The discovery of invertebrate LGICs such as the nematode GluCl^1^,^2^ extended the ion selectivity of glutamate-gated ion channels to Cl^-^, while prokaryotic LGICs extended the repertoire of endogenous extracellular ligand to protons^3^,^4^. The physiological role of those prokaryotic LGICs in unicellular organisms is not yet clearly understood. In vertebrate, activation of anionic LGICs such as the GABA*A* and glycine receptors generates an inward flow of chloride ions, hyperpolarizing the cell and inhibiting the action potential transmission in the nervous system, while activation of cationic LGICs depolarizes the cell for propagating the action potential. The ion selectivity of cationic LGICs is less stringent than the anionic ones, as they are permeable to both Na^+^ and K^+^ and for some to Ca^2+^. To our knowledge, only one potassium-selective LGIC has been reported, the prokaryotic glutamate receptor GluR0 from *Synechocystis*^5^.

With the initial objective of designing versatile biosensors for various ligands, we created artificial LGICs by fusing metabotropic receptors (G protein-coupled receptors, GPCRs) to a mammalian potassium-selective channel (Kir6.2). In these fusion proteins called ion channel-coupled receptors (ICCRs), Kir6.2 gating is controlled by the GPCR conformational changes upon ligand binding. ICCRs have not only the potential to extend the specificity of recognized ligands to the large repertoire of GPCR ligands, but also exhibit potassium selectivity through Kir6.2. Kir6.2 is the pore-forming subunit of natural ATP-sensitive potassium (K_ATP_) channels composed of a homotetrameric pore of Kir6.2 surrounded by four sulfonylurea receptors (SUR). Those channels act as cellular metabolic sensors, converting variations of the intracellular ADP/ATP ratio into changes of the membrane potential. Kir6.2 hosts inhibitory binding sites for intracellular ATP and displays, within ICCRs, an IC_50_ in the range of hundred micromolars^6^ leading to a partially opened channel at rest in a cellular environment. This basal activity allows the observation of the channel closing or opening evoked by GPCR ligands. These two opposite regulations have been observed in the first ICCRs composed of either the human muscarinic M2 receptor, which opened the channel in presence of acetylcholine, or the human dopaminergic D2_L_ receptor (D2), which closed the channel in presence of dopamine^6^ (Fig. 1a). The rationale of the present work was to understand how these two receptors, from the same GPCR subfamily (Class A) and preferentially coupled to the same heterotrimeric G proteins (Gi/o), regulate the channel gating in opposite directions when they adopt an agonist-bound state.

To address this question, we postulated that M2 and D2 receptors undergo similar agonist-induced conformational changes, as observed in several crystallographic structures of class A GPCRs in their active states^7^,^8^,^9^,^10^,^11^,^12^,^13^,^14^,^15^,^16^,^17^,^18^,^19^,^20^, and hypothesized that sequence differences in the ICCR region linking the receptor and the channel influenced the receptor-mediated regulation of Kir6.2 gating. The alignment of the C-termini of M2 and D2 shows that the M2 C-terminus is 9 residues longer than the D2 C-terminus (Fig. 1b). Using a protein engineering approach, we investigated whether this difference in the GPCR C-terminus length is involved in the opposite regulation of the Kir6.2 gating.

Our results not only demonstrate the role of the GPCR C-termini in the gating regulation, but also define the means to finely tune Kir6.2 gating by the fused GPCRs. Our work demonstrates that artificial potassium-selective LGICs can be engineered to feature adjustable and versatile responses to natural ligands and opens the way for new applications in synthetic biology.

## Results

### The regulation of Kir6.2 gating is inverted by truncation of the M2 C-terminus

**Figure 2:**
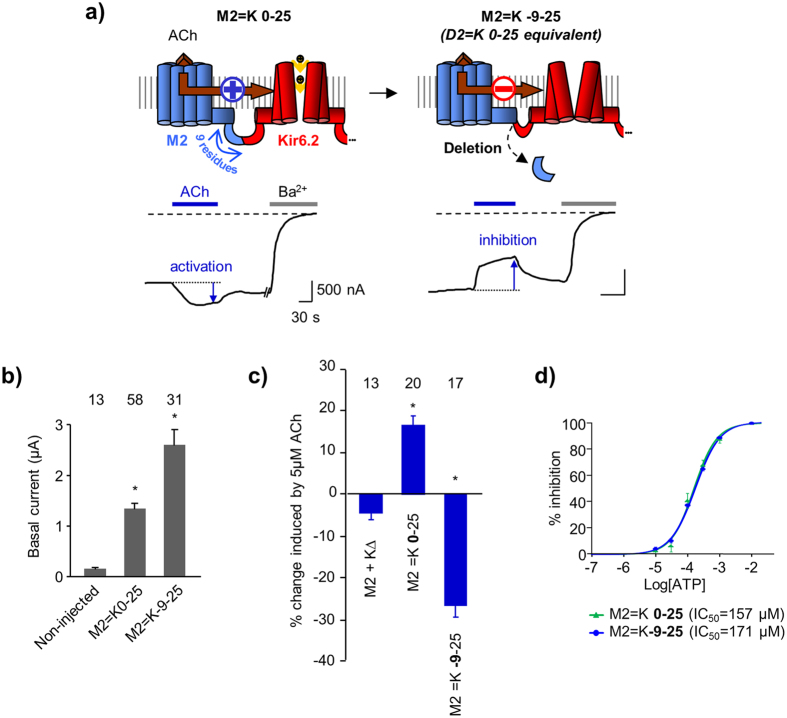
Shortening the M2 C-terminus inverts the gating regulation. (**a**) M2 = K0-25 designates 0 residue deleted from the M2 C-terminus and 25 residues deleted from the Kir6.2 N-terminus. Under the diagram is shown a representative Two-Electrode Voltage-Clamp (TEVC) recording of the ICCR heterologously expressed in *Xenopus* oocytes at −50 mV in symmetrical K^ + ^concentration. ACh: 5 μM Acetylcholine. Ba^2 + ^(3 mM) is a blocker of potassium channels. Deletion of 9 residues at the M2 C-terminus (M2 = K-9-25) inverted the regulation of the channel. (**b**) Basal currents showing significant expression of the muscarinic ICCRs. Histogram shows mean ± s.e.m. with numbers of experiments above bars. * indicates a significant difference (P < 10^−9^) from non-injected oocytes. (**c**) Average in % of current amplitude ± s.e.m. induced by 5 μM ACh. Positive and negative values reflect opening and closing of the channel respectively. M2 + Kir6.2Δ: coexpression of M2 with Kir6.2 truncated of its last 36 residues. Number above bars = number of experiments (n). *: significant difference (P < 10^−7^) from M2 + KΔ. (**d**) ATP concentration-effect curves of M2 = K0-25 (control) and M2 = K-9-25 obtained by application of increasing concentrations of ATP to the cytoplasmic face of excised inside-out patches. ATP is an endogenous blocker of the channel by direct binding to Kir6.2.

To determine whether the difference in the M2 and D2 C-termini is implicated in the opposite regulation of the channel gating, we first removed the last 9 residues of the M2 receptor to match the D2 C-terminus length (Fig. 2a). We used the following nomenclature: M2 = K-9-25 designates the fusion of M2 and Kir6.2 (K) after deletion of the last 9 residues of the C-terminus of M2 and deletion of the first 25 residues from the N-terminus of Kir6.2.

Functional characterization was performed with the two-electrode voltage-clamp (TEVC) technique on *Xenopus* oocytes heterologously expressing the ICCRs. We used the basal current generated by the fused Kir6.2 as a reporter of the expression of M2 = K-9-25 at the plasma membrane. Figure 2b shows a basal current 16-fold higher for M2 = K-9-25 than for the non-injected oocytes, indicating a significant surface expression of the ICCR. To assess the regulation of Kir6.2 by the C-terminally truncated M2, we examined the response of the ICCR to the application of 5 μM acetylcholine (ACh), the endogenous M2 agonist. TEVC recordings of M2 = K-9-25 clearly showed that ACh decreased Kir6.2 channel activity while, for the full length M2-based ICCR (M2 = K0-25) it augmented it (Fig. 2c). Consequently, the truncation of the last 9 residues of M2 inverted the regulation of the gating. The role of the M2 C-terminus is either direct, in the transduction pathway of conformational changes from the M2 ligand-binding site to the Kir6.2 gate(s), or indirect, affecting the intrinsic activity of the two fused proteins or their interactions outside the linker region.

In order to investigate this latter hypothesis, we controlled the intrinsic activity of the two fused proteins by measuring the ATP sensitivity of Kir6.2 and the activation of G proteins by M2.

Kir6.2 is blocked by intracellular ATP with an IC_50_ of ~100 μM when expressed alone^21^ or in ICCR^6^. ATP sensitivity is a reliable indicator of Kir6.2 activity as alterations of the gating affect the apparent affinity to ATP. Using the inside-out patch-clamp technique, we measured the ATP concentration-effect relationship of M2 = K-9-25 and found that it is identical to that of M2 = K0-25 (Fig. 2d), indicating that truncation of the M2 C-terminus does not affect the gating properties of Kir6.2.

**Figure 3:**
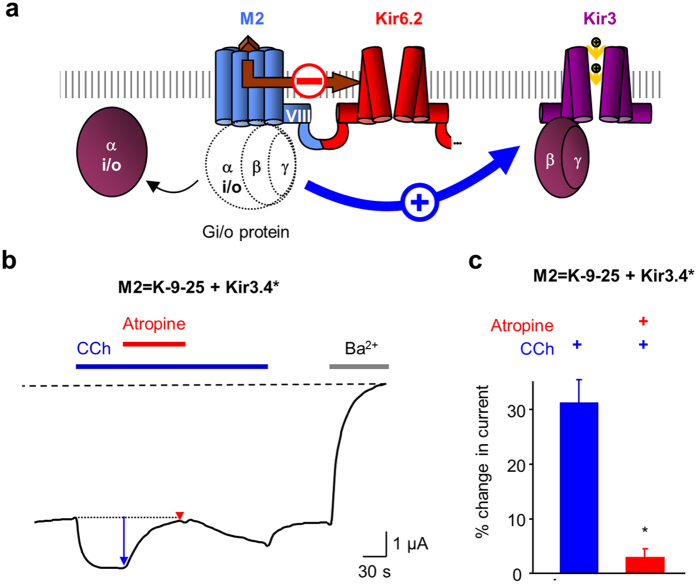
Engineered M2 receptors retain the capacity to activate Gi/o proteins. (**a**) Diagram of the functional assay of G protein activation in *Xenopus* oocytes using the G protein-activated Kir3 channels. mRNA coding for the ICCR and the Kir3 channel are co-injected in *Xenopus* oocytes. Binding of acetylcholine onto M2 triggers activation of the endogenous Gi/o proteins. The release of the Gβγ subunits leads to opening of Kir3 channels. The current generated by the Kir3 channels being several-fold higher than that of the current generated by Kir6.2 in the ICCR, the inhibitory effect of the agonist on M2 = K-9-25 is masked by the activating effect on Kir3. (**b**) Representative TEVC recording from an oocyte co-expressing M2 = K-9-25 and Kir3.4* showing activation of the Kir3 channels by carbachol (CCh, 5 μM) (blue arrow) and the antagonist effect of 1 μM atropine (red arrow). (**c**) Average current change induced by 5 μM CCh (blue bar) and 5 μM CCh + 1 μM atropine (red bar). *: P = 4.10^−3^ between the two bars. n = 4.

To evaluate the activity of the truncated M2 receptor, we used Kir3 channels as reporters of the activation of Gi/o proteins (Fig. 3a). Physiologically, binding of the neurotransmitter ACh to M2 receptors triggers the activation of heterotrimeric Gi/o proteins, leading to the activation of G protein-activated potassium channels (Kir3) by the Gβγ subunits. We co-expressed M2 = K-9-25 with a mutated Kir3 channel (Kir3.4*) able to form high-activity homotetramers^22^. Since the Kir3.4* channels exhibit a larger basal current ( > 2-fold) than the fused Kir6.2, the change in recorded potassium current is predominantly generated by the Kir3 channels and distinctly reports Gi/o protein activation. Application of 5 μM carbachol (CCh), a synthetic muscarinic ligand, clearly activated Kir3.4* (Fig. 3b), demonstrating that the truncated M2 receptor, within the ICCR, retains the ability to activate Gi/o proteins. As expected Kir3.4* activation is abolished by the M2 antagonist atropine. These results demonstrate that the ability of M2 to bind ligands and to activate G proteins is not impaired by truncation of its C-terminus within the ICCR.

Therefore, the intrinsic activities of Kir6.2 and M2 appear unaltered in M2 = K-9-25. These results reinforce the hypothesis of a direct role of the M2 C-terminus in the inversion of the gating.

### Extending the D2 C-terminus also inverts the gating

**Figure 4:**
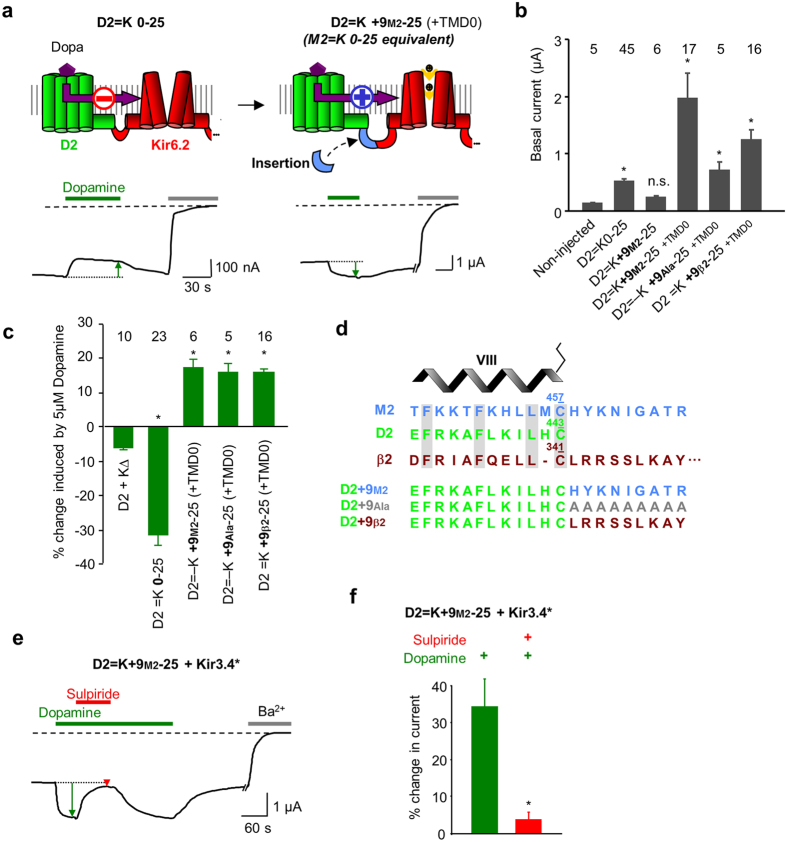
Extending the D2 C-terminus also inverts the gating regulation. (**a**) D2 = K0-25 is inhibited by 5 μM dopamine (Dopa). D2=K+9M2-25 contains an extended D2 C-terminus with the last 9 residues from M2 and is activated by 5 μM Dopamine. The first transmembrane domain (TMD0) of SUR1 is co-expressed for boosting the surface expression of the ICCR. (**b**) Basal currents of dopaminergic ICCRs show lack of surface expression of D2=K+9M2-25. Co-expression with the N-terminal transmembrane domain of SUR1 (TMD0) restores the surface expression. Extended C-terminus of D2 with 9 alanines is noted D2=K+9Ala-25 and with 9 C-terminal residues from the human β2 adrenergic receptor is noted D2=K+9β2-25. *: compared to Non-Injected P < 0.02; ns (not significant) P = 6.10^−2^. (**c**) Average in % of current amplitude ± s.e.m. induced by 5 μM dopamine. *: P < 10^−3^ (ref.: D2 + K∆). (**d**) Alignment of the C-terminal sequences of the human receptors M2 (blue), D2 (green) and β2 adrenergic (brown) receptors. The position of the helix VIII is shown above the sequences and the terminal cysteines are numbered and depicted as palmitoylated. The long C-terminus of the β2 adrenergic receptor is partially represented. The 3 different extensions of the D2 C-terminus are shown in the lower panel. (**e**) TEVC recording of D2=K+9M2-25 + Kir3.4* showing D2-mediated activation of Kir3.4* channel by 5 μM dopamine (green arrow) and the antagonism (red arrow) by 5 μM sulpiride. (**f** ) Average current change induced by 5 μM dopamine (green bar) and 5 μM dopamine + 5 μM sulpiride (red bar) on D2=K+9M2-25 + Kir3.4*. * P = 9.10^−3^ between the two bars. n = 6.

To confirm the role of the GPCR C-terminus in the regulation of Kir6.2 gating, we designed the reciprocal construct by extending the D2 C-terminus with the last 9 residues from M2 (D2 = K + 9_M2_-25) (Fig. 4a). The surface expression of D2=K+9M2-25 was not detectable, the basal current being similar to that of non-injected oocytes (Fig. 4b). This lack of surface expression has been already observed with β2-adrenergic^23^ and the opsin^24^ ICCRs and was solved by co-expression with the first transmembrane domain (TMD0) of the sulfonylurea receptor SUR1^25^ which interacts spontaneously with Kir6.2. For D2 = K + 9_M2_-25, the co-expression with TMD0 also boosted its surface expression with an 8-fold increase in the average basal current amplitude (Fig. 4b), allowing its functional characterization.

To assess the regulation of Kir6.2 by the C-terminally extended D2 receptor, we tested the effect of the endogenous agonist dopamine on *Xenopus* oocytes co-expressing D2 = K + 9_M2_-25 and TMD0. We observed that the extended dopaminergic ICCR can be activated by the agonist, indicating that the inversion of the gating occurred also reciprocally (Fig. 4c). To confirm that this effect is due to the length of the C-terminus and not to the nature of the inserted amino acids, we replaced the M2 C-terminal sequence by 9 alanines (D2 = K + 9_Ala_-25) or 9 residues from the β2-adrenergic receptor C-terminus (D2 = K + 9_β2_-25, alignment shown in Fig. 4d). For both constructs, the channel was activated by dopamine with the same amplitudes as the ones observed for D2 = K + 9_M2_-25 (Fig. 4c). These results confirm that the gating inversion is due to the C-terminal extension of the D2-based ICCRs in a sequence-independent manner.

As for the modified M2-based ICCR, we also controlled that the activity of the D2 receptor was unaffected by its C-terminal extension using co-expression of D2 = K + 9_M2_-25 with the G protein-activated Kir3.4* channel. The activation of Kir3.4* by dopamine and its antagonism by the D2 antagonist sulpiride confirmed the functional integrity of the extended receptor within D2 = K + 9_M2_-25 (Fig. 4e,f).

Consequently, permutation of the M2 and D2 C-termini inverted the gating without affecting the intrinsic activities of the receptors and the channel.

### Inversion of the regulation does not affect the ICCR pharmacological properties

**Figure 5:**
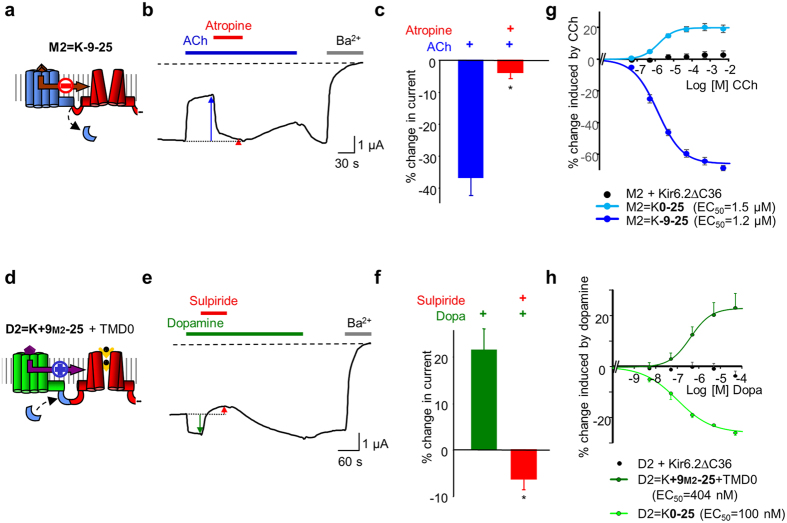
The C-terminally altered M2 and D2 ICCRs retain their pharmacological properties. (**a**) Diagram of the M2 = K-9-25 ICCR. (**b**) Representative TEVC recording of M2 = K-9-25 showing 5 μM ACh-evoked inhibition (blue arrow) and the antagonist effect of 1 μM atropine (red arrow). (**c**) Average current change induced by ACh 5 μM (in blue) and ACh 5 μM + Atropine 1 μM (in red) in oocytes expressing M2 = K-9-25. *: P = 3.10^−4^ (ref: Ach). n = 8. (**d**) Diagram of the D2 = K + 9M2-25 ICCR. (**e**) TEVC recording showing activation of D2=K+9M2-25 + TMD0 by 5 μM dopamine (green arrow) and its antagonism (red arrow) by 5 μM sulpiride. (**f**) Average effects of dopamine and sulpiride on D2=K+9M2-25 + TMD0. *: P = 1.10^−3^ (ref.: dopamine). n = 5. (**g**) Concentration-effect curve of carbachol (CCh) of the indicated constructs. Each point is an average of 8 to 23 measurements. (**h**) Concentration-effect curves of dopamine of the indicated constructs. n = 4.

We controlled, as well, that the pharmacological properties of the modified ICCRs that displayed inverted gating were not altered. As shown previously, the engineered ICCRs kept their sensitivity to competitive antagonists (Fig. 5a–f). Furthermore, the ligand-concentration dependence of the engineered ICCRs was assessed by sequential applications of increasing concentrations of agonists on single *Xenopus* oocytes and repeated on different oocytes from different batches. We observed that the ICCRs still display a ligand concentration-dependent signal even though the channel gating is inverted (Fig. 5g,h). Interestingly, the modification of the GPCR C-termini did not notably change the apparent affinity for agonists. This indicates that modifying the length of the GPCR C-terminus does not alter the ligand binding properties and the subsequent agonist-induced conformational changes of the GPCR.

### The amplitude and sign of the gating correlate with the M2 C-terminus length

**Figure 6:**
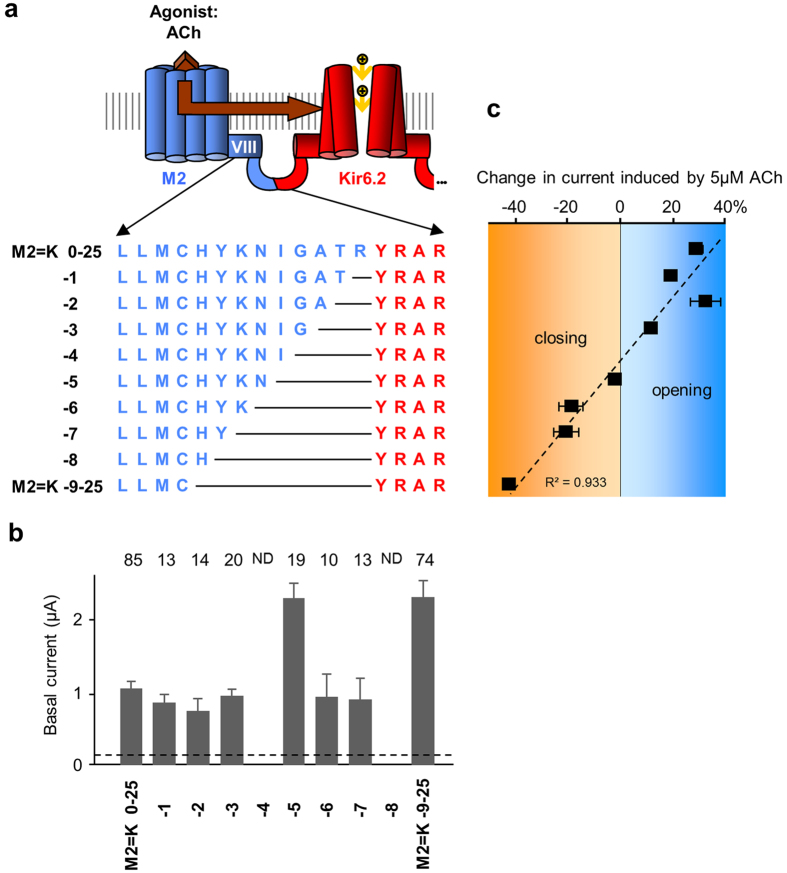
The gating regulation of M2 = K is correlated with the length of the receptor C-terminus. (**a**) Sequences of the fusion zone of M2 = K ICCRs with incremental deletions in the M2 C-terminus (in blue). (**b**) With 2 exceptions, all M2 C-terminally truncated ICCRs are expressed into the oocyte plasma membrane. ND: Not Determined because of failure of their genetic engineering. *: significantly expressed compared to non-injected oocytes P < 10^−2^. (**c**) Average effects of 5 μM ACh on each construct. Activation is positive, inhibition negative. Each point was determined from 8 experiments or more. The dashed line represents the best linear correlation fit.

Deleting or adding 9 residues at the GPCR C-terminus totally reverses the regulation of the gating confirming the crucial role of this domain in the regulation of the channel. However, the molecular mechanisms involved are not clearly identified. To further investigate the role of the GPCR C-terminus at a single residue resolution, we incrementally truncated the M2 C-terminus residue by residue from 0 to -9 (Fig. 6a). This approach could provide indications on the secondary structures of this domain that is unresolved in the published crystal structures of M2^17^,^26^. A periodicity of 3-4 residues or 2 residues would reveal the presence of a α-helix or a β-sheet, respectively.

Except for the constructs M2 = K-4-25 and M2 = K-8-25 that we were unable to obtain despite intensive efforts, all truncated ICCRs were engineered and their surface expression was sufficient for functional characterization (Fig. 6b).

The response of the channel in the truncated ICCRs was assessed by application of 5 μM ACh. The gradual truncation of the M2 C-terminus resulted in a progressive downward evolution of the ligand-induced response from opening to closing that followed a linear trend (Fig. 6c). This observation, which provided no evidence for secondary structures, confirmed the direct implication of the receptor C-terminus length in the gating of Kir6.2. The progressive inversion of the gating suggests a repositioning of the receptor with respect to Kir6.2, from an optimal position for opening to an optimal position for closing it.

## Discussion

The initial objective of this study was to identify the molecular determinants at the origin of the opposite regulation of the Kir6.2 gating by the fused M2 and D2 receptors. By swapping the M2 and D2 C-termini, we created ion channels with inverted GPCR-evoked regulation. Thus, the same receptor can regulate the ion channel in opposite direction when its C-terminus is engineered. The modified receptors activate G proteins indicating that ligand-induced conformational changes are unaffected by the C-terminal modifications. Therefore the opposite regulation of the ion channel is not related to different conformational changes of the receptors but to the length of the receptor C-terminus. The observation of a linear correlation between the M2 C-terminus length and the regulation of the channel raised the hypothesis that progressive truncations of the M2 C-terminus bring the two proteins closer with reorientation of the receptor. The presence of a pivot between ligand-activated and inhibited constructs suggests that the receptor would tilt around a specific position, depending on the length of the GPCR C-terminus. Receptors with long C-terminus would adopt a position relative to the ion channel that induces opening upon ligand binding, while receptors with short C-terminus would change their position and trigger a closing. The Supplementary Figure S1 depicts how receptors with similar conformational changes could generate opposite regulation of the fused ion channel.

This work revealed the prevalent role of the GPCR C-terminus in the regulation of Kir6.2 gating within ICCRs. It also demonstrated how to finely tune the gating of Kir6.2, in terms of amplitude and direction of the regulation, by modifying few residues in the C-terminus of the M2 and D2 receptors. Original applications of these artificial LGICs are envisioned in the field of synthetic biology especially in the development of customized cell responses and the minimization of the genome of synthetic organisms.

In the customization of cell response, these engineered ICCRs constitute artificial LGICs able to hyperpolarize or depolarize the cell membrane with different amplitudes in response to the endogenous agonists acetylcholine and dopamine. While natural LGICs rapidly desensitize, the response of ICCRs is sustained over several minutes. In physiological conditions, activation of M2 and D2 by endogenous ligands induces a hyperpolarization mediated by the opening of the G protein-activated Kir3 channels. Agonist-blocked M2 and D2 ICCRs could be used to invert this response because they offer the ability to depolarize the cell membrane either by endogenous ligands, or by exogenous pharmacological agonists. ICCRs being independent of intracellular signalling^27^, they have the capacity to modulate the membrane potential of any cell with different expression pattern of G protein subtypes and even no expression of G proteins. ICCR could be also used to restore K^+^ uptake in cells like the *Saccharomyces cerevisiae* strain lacking the K^+^ transporter Trk1p and Trk2p for screening purposes^28^. An exception are the prokaryotic cells which do not contain the phosphatidylinositol 4,5-bisphosphate (PIP2) lipid required for the activity of mammalian Kir channels like Kir6.2. Exogenous or modified membrane proteins are often poorly expressed at the plasma membrane. In some cells, surface expression of ICCRs could require the deletion of an endoplasmic reticulum retention signal in the channel C-terminus (ΔC26 to ΔC36)^21^,^29^,^30^. This manoeuvre was not required in *Xenopus* oocytes, except for ICCRs with an engineered 3^rd^ intracellular loop occluding the G protein binding site^27^,^31^.

To reduce background K^+^ currents and increase the amplitude of current change generated by ICCRs, optimal conditions would be synthetic biology systems designed without endogenous K^+^ channels. However, leak potassium channels are vital for numerous cells to maintain a negative resting membrane potential. In such cells, expression of ICCR as unique potassium channels is made possible thanks to the basal activity of the fused Kir6.2 that substitutes the activity of the endogenous leak channels.

## Conclusion

This study demonstrats the role of the M2 and D2 C-terminus length in the regulation of the Kir6.2 gating within ICCRs. It provides the instructions on how to finely tune the channel to adjust the amplitude and sign of the regulation induced by M2 and D2 agonists. These artificial ligand-gated ion channels constitute therefore a unique tool for synthetic biology applications in chemogenetics^32^ with the properties of potassium selectivity, independence of intracellular signalling and inverted signal to physiological conditions.

## Methods

### Materials

All chemicals were purchased from Sigma-Aldrich. Stock solutions were: acetylcholine chloride 5 mM (water); carbachol chloride 250 mM (water); atropine 1 mM (ethanol); dopamine hydrochloride 5 mM (water); (S)- (−)-sulpiride 5 mM (DMSO).

### Genetic engineering

All genes were in pGEMHE-derived vectors optimized for protein expression in *Xenopus* oocytes. The template M2 = K0-25 and D2 = K0-25 were created in a previous study by two-step PCR as previously described^6^,^31^. Truncations of the M2 C-terminus were performed by one-step PCR with the QuikChange site-directed mutagenesis kit (Agilent) using primers overlapping the deleted sequence. For D2 C-terminus extension, asymmetric primers were designed as hybrid sequences with the inserted sequence (M2 C-terminus) in 5′ and the template sequence in 3′ 33 Positive clones were verified by sequencing. DNA was amplified using Qiagen MidiPrep Kit, linearized in the 3′ region of the polyA tail, purified by standard phenol:chloroform extraction and transcribed in mRNA using the T7 mMessage mMachine Kit (Thermo Fisher Scientific). mRNAs were purified by the classic phenol:chloroform protocol, analysed by agarose-gel electrophoresis and quantified by spectrophotometry^34^. The same protocols were used for the hamster TMD0(SUR1)-F195^25^ and the mutated channel Kir3.4_S143T_, designated Kir3.4^* ^22.

### Electrophysiological recordings

*Xenopus* oocytes were prepared as previously reported^6^,^31^. Animal handling and experiments fully conformed with European regulations and were approved by the Ethics Committee of the Commissariat à l′Energie Atomique et aux Energies Alternatives (Ethics Approval #12-040 to CJM). Authorization of the animal facility has been delivered by the Prefect of Isere (Authorization # D 38 185 10 001).

Briefly, after surgical retrieval, the oocytes are defolliculated by type 1 A collagenase. Each oocyte was injected with 50 nl of RNAse-free water containing the desired mRNAs. The amounts used per oocyte were: ICCR 4.3 ng, TMD0 1 ng, Kir3.4* 2.5 ng. Microinjected oocytes were incubated for more than 2 days at 19 °C in Barth′s solution (in mM: 1 KCl, 0.82 MgSO_4_, 88 NaCl, 2.4 NaHCO_3_, 0.41 CaCl_2_, 16 Hepes, pH 7.4) supplemented with 100 U.ml^−1^ of penicillin and streptomycin and 0.1 mg.ml^−1^ of gentamycin. Whole-cell currents were recorded with the two-electrode voltage clamp (TEVC) technique. Microelectrodes were filled with 3 M KCl and oocytes were bathed in the following solution (TEVC bath, in mM): 91 KCl, 1.8 CaCl_2_, 1 MgCl_2_, 5 HEPES, 0.3 niflumic acid (to block endogenous Cl^-^ currents), pH 7.4. The TEVC voltage protocol consisted of 500 ms steps to -50, 0 and + 50 mV, during which current was measured, separated by 5 s at a holding potential of 0 mV. The values shown in the figures are those recorded at −50 mV.

Average values are presented as mean ± s.e.m. Ba^2+^ (3 mM) was used as a generic potassium channel blocker to establish the amplitude of potassium currents. Change of potassium current induced by ligand was calculated with the basal current as reference.

Excised inside-out patch-clamp experiments were performed as previously described^35^ in symmetrical K^ + ^concentration (150 mM) and at −50 mV.

Negative controls were performed with the unfused proteins M2 + KΔC36 and D2 + KΔC36. In those controls, Kir6.2 is truncated of its last 36 residues (KΔC36) in order to remove a known endoplasmic reticulum retention signal and to allow the surface expression of the channel alone^21^.

## Additional Information

**How to cite this article**: Moreau, C. J. *et al*. Tuning the allosteric regulation of artificial muscarinic and dopaminergic ligand-gated potassium channels by protein engineering of G protein-coupled receptors. *Sci. Rep.* **7**, 41154; doi: 10.1038/srep41154 (2017).

**Publisher's note:** Springer Nature remains neutral with regard to jurisdictional claims in published maps and institutional affiliations.

## Supplementary Material

Supplementary Information

## Figures and Tables

**Figure 1:**
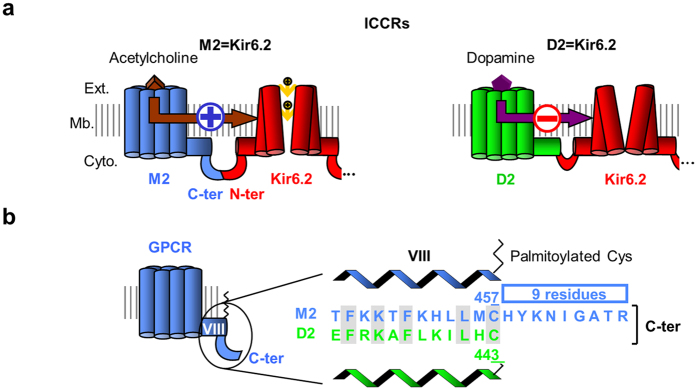
Opposite regulation of muscarinic and dopaminergic Ion Channel-Coupled Receptors (ICCRs). (**a**) The artificial Ion Channel-Coupled Receptors are created by fusion of the Kir6.2 N-terminus to the C-terminus of G Protein-Coupled Receptors (GPCRs). Only one full-length subunit and the channel of a second subunit are shown. In M2 ICCRS, agonists upregulate Kir6.2 while in D2 ICCRs, they downregulate. Ext.: extracellular side, Mb.: membrane, Cyto.: cytoplasmic side. (**b**) Alignment of the C-termini of M2 and D2 receptors.
