# Supplementary material for: Tuning the allosteric regulation of artificial muscarinic and dopaminergic ligand-gated potassium channels by protein engineering of G protein-coupled receptors
Source: Sci Rep. 2017 Feb 1;7:41154. doi: 10.1038/srep41154 (PMC5286527; doi:10.1038/srep41154)
Supplement: Supplementary Information [file srep41154-s1.pdf]

Supplementary information

Tuning the allosteric regulation of artificial muscarinic and dopaminergic ligand-gated potassium channels by protein engineering of G protein-coupled receptors

Christophe J. Moreau*, Jean Revilloud, Lydia N. Caro, Julien P. Dupuis, Amandine Trouchet, Argel Estrada-Mondragón, Katarzyna Nieścierowicz & Michel Vivaudou.

Supplementary Figure-S1 (Moreau)

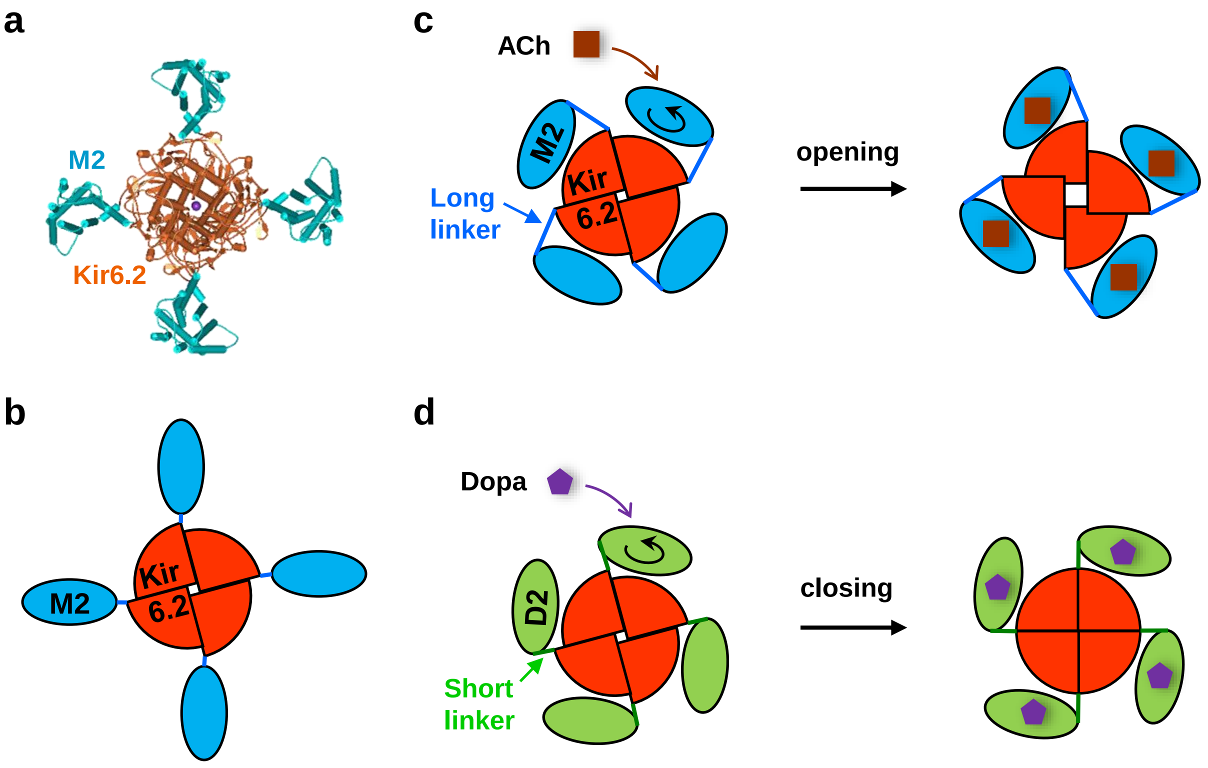

Supplementary Figure S1. Model of the opposite regulation of Kir6.2 by M2 and D2. (**a**) Top extracellular view of a muscarinic ICCR model with arbitrary positions for the receptor relative to the pore. M2 receptor is in cyan and Kir6.2 in orange. (**b**) Schematic representations of the muscarinic ICCR with equivalent positions of proteins to the panel a. (**c**) Hypothetic mechanism of ligand-induced activation of M2-K0-25. Conformational changes depicted by the curved black arrow stabilize the channel in open state. ACh stands for acetylcholine. (**d**) With a shorter linker, the D2 dopaminergic receptor could be oriented differently in D2-K0-25. Thus similar conformational changes than those occurring in M2 would stabilize a closed-state of the channel in the dopaminergic ICCRs.
